# The Hygiene of the Mind1President’s address at the thirtieth annual meeting of the Medical Association of Central New York, at Buffalo, October 18, 1897.

**Published:** 1897-11

**Authors:** Edward B. Angell

**Affiliations:** Rochester, N. Y., Member of the American Neurological Association; neurologist to the Rochester City Hospital, etc.


**BUFFALO MEDICAL JOURNAL.**

Vol. XXXVII. NOVEMBER, 1897. No. 4.

### Original Communications.

### THE HYGIENE OF THE MIND.[1]

By EDWARD B. ANGELL, M. D., Rochester, N. Y.,
Member of the American Neurological Association ; neurologist to the Rochester City Hospital, etc.

WHEN Harvey, by his brilliant discovery of the circulation of the blood, nearly three centuries ago, laid the foundation for investigation upon which modern medicine has reared so noble a structure, medical thought was dominated by the mysticism and supernaturalism of the middle ages.

Since his time, in the study of the human body, patient, scientific observation has supplanted medieval theory. But to the investigation of mental phenomena how halting have we been in the application of the scientific method ! Within the memory of practising physicians of today the study of the human mind was relegated to the metaphysician or theologian, whose knowledge was largely speculative or *a priori*.

Today, it is safe to say, it is no longer regarded as evidence of heresy to proclaim that the manifestation of mind depends upon the molecular structure and metabolic activity of the brain ; if, indeed, mind be not the function of that organ. The science of mind, then, must take its place with all other sciences and must result from the patient study and classification of its phenomena upon the well-established principles of inductive reasoning.

The study of mind and its manifestation, from the standpoint of the physician, has largely been limited in the past to its perverted action through disease. Our knowledge of the minute

1. President's address at the thirtieth annual meeting of the Medical Association of Central New York, at Buffalo, October 19, 1897.

anatomy of the brain, of the functional specialisation of certain centers, is so recent and still so incomplete, that the field has been far from attractive and as yet barren of results.

The painstaking investigation of psychological phenomena is today as necessary and useful to the physician as the detailed study of the phenomena of the physical life. And, curiously, the scientific investigation of hypnotism marks the beginning of the application of the scientific method to the study of mind. As Wyllys Taylor[1] well says:

> The significance of the study of hypnotism lies not so much in the fact that this or that person has been benefited by its use, as in the fact that phenomena relating to the mind have at last gained recognition among medical men on precisely the same grounds as phenomena in the physical sphere. A scientific psychology, based on observation, has come to be regarded as a legitimate and practical study for the physician as well as for the special student. The hazy conceptions, so long surrounding all facts of the mental life, are gradually disappearing, and men are beginning to realise that however complex and difficult of apprehension such facts may be, there is at least no scientific heresy in their thorough investigation by such means as we have at our disposal.

However we may regard the vexed question of body and mind, we at least are justified, from every physiological analogy, in assuming that mind manifests itself through brain activity and through brain activity alone. It depends for its highest expression upon a perfectly developed brain in perfect action.

The science of neurology has of recent years done much to advance our knowledge of this relationship. Its anatomical and physiological investigations, as well as its pathological and clinical observations, have contributed largely to a clearer comprehension of the problem. I only need call attention to the advance made in localisation of cerebral action, such as the so-called motor zone and the visual and auditory centers with their connecting tracts. More recently the identification of the neuron as a distinct entity is a triumph of patient investigation and will lead to a clearer understanding of brain processes in time. Furthermore, the evidence we have that muscular activity directly influences the nutrition of its corresponding center in the motor zone is a physiological observation of much importance in the question of brain development. Through proper stimuli other cerebral areas, auditory,

1. Taylor—The Mental Element in the Treatment of Disease, p. 13.

visual or speech centers, can be developed and their connecting tracts made more impressionable.

The first effort of education in the child should be the stimulation and exercise of the various senses. Observation naturally plays an important part and is one of the earliest faculties developed in the growing infant. Maudsley[1] insists strenuously upon the great value of the natural sciences in the early training of the mind, an observation which tallies well with the proved value of the nature studies of the kindergarten. The power of attention, the great disciplinary engine of the mind, is more readily acquired through the study of concrete things. The value of this in mental development I need scarcely mention, for is it not true that the capacity of the individual mind is largely measured by the power of sustained attention?

Undoubtedly muscular activity has a direct effect upon brain development. Not only does it react upon the so-called motor convolutions, but neighboring centers are influenced as well. But the manual training school or mechanical instruction is of more real value than mere gymnastics. Manual dexterity has a definite bearing in stimulating mental coördination. It is further true that physical effort with a definite purpose in view is of more worth to brain development than meaningless muscular activity.

In all educational processes the temperament of the individual must be considered and dealt with accordingly. The sanguine and choleric need less stimulation than the phlegmatic and melancholic.

Education of the young in its truest sense, then, must mean adequate development of the brain, not merely the training of the mind, or the acquisition of knowledge. Without doubt its narrower limitation of meaning is due largely to the fact that education in the past has been under the control of the clerical or metaphysical mind. Education can never become scientific until it is founded upon physiological data; it should have for its aim the full development of the individual; it should be physical, not metaphysical; it should be complete, not partial. If the brain is thus developed, mental action will be normal, the acquisition of knowledge rapid and the judgment well balanced.

Turning now from this broad subject of general education, let us consider that which more directly concerns us as physicians, the preventable causes of mental deterioration. "*Facilis descensus Averni*," wrote the Latin poet. In human development, reversion

1. Maudsley—Responsibility in Mental Disease, p. 301.

to a lower type is as easy. Ages were required for the evolution of the brain of today; generations suffice to reduce it to a condition even lower than that of the ape. Degeneration is our mental hell, self-indulgence its pathway, discipline and education our only mode of escape.

It is not within the scope of this address to discuss the physical defects underlying mental degeneracy. They are well enough known to every student of medicine. It must suffice to call attention to their influence in absolutely limiting mental development. They may be the hidden birth-marks of heredity, the defects of arrested development, the ravages due to organic disease or the toxic influence of alcoholism. Among less common causes of mental obtuseness in children may be mentioned deafness, nasal obstruction, limited patches of meningeal inflammation following infectious diseases and similar specific conditions, which should be remembered when a practitioner is consulted in regard to a backward or refractory child. Peevishness and disobedience in children are often due to mental irritability from maldigestion, appropriate treatment of which is far more efficient as a moral agency than the much-vaunted rod. Even in such instances special treatment or a special mode of education may do much to overcome organic defects in brain organisation.

The influence of bodily health upon mental action is so well known that a discussion of its absolute importance need not detain us. Who has not met the depression, gloom and apprehension due to a perverted liver action? Timidity and panic often are the significant indications of a disturbed innervation of the heart. Less commonly, we note the baneful effects upon the mind wrought by the deficiency of special organic secretions. The idiocy of the creatin, the depravity of the eunuch, the sluggishness of acromegaly are instances in point.

In addition to these cases of mental deficiency, due to defective development, structural change or toxic influences, we recognise a large group of so-called degenerates, unfortunate dwellers on the borderland of insanity, for whose mental salvation much may be done by judicious guidance. The enormous exaltation of self-consciousness, the "subjective ego," is the characteristic perversion of the mental action. Through faulty education and early fostering of self-feeling, the mind becomes unduly sensitive to subjective impressions and ideas. Gradually, mental attention, fixed more and more upon subjective impressions, loses its true quality—that of determining and directing outgoing impulses in the accomplishment of objective results. The undue exaltation of self-consciousness often is one of the serious results of neurasthenic conditions. Fostered by anxious sympathy of friends, disturbed by strange sensations, self-introspection is enormously developed and mental action is but the automatic by-play of morbidly exaggerated sense impressions. In the management of this type of morbid mind, denial of the existence of such sensations is useless. The principle of diversion of the attention is our strongest resource. Morbid sensations may be replaced by real ones, hurtful sympathy may be repressed and through a constant daily routine of conscious effort, normal mental action reëstablished. In this connection, it is of importance to discriminate between subjective and objective fears. The one rarely causes serious results, the latter often is attended by evil consequences, fear resulting from subjective impressions not doing serious harm and rarely needing contradiction. When this tendency to undue sensory excitability is early recognised in a growing child much may be done to prevent its development by consistent application of these principles.

Another danger to which the mind is subject comes from the predominating influence of a fixed idea. Continued attention to any one line of thought, to one mode of mental activity, may result in enchaining the attention itself. It is important to break the continuity of thought frequently and at least for a brief period to divert the attention into other channels. As an illustration of how strong the tendency of the motor apparatus of the mind is to execute the behests of fixed attention, call to mind the absolute certainty with which a beginner with the bicycle runs against the very obstruction which he would most sedulously avoid. His whole attention is fastened with dread upon the obstacle and the muscles but execute the dominating impulse. Without doubt the awful dread that takes entire possession of the mind of one standing on Niagara's brink many times has inspired the leap which is wrongfully ascribed to suicidal motive.

The hurry of modern civilisation is detrimental to conservation of brain power, the habit of rapid discharge of nerve energy hostile to normal inhibitory control. There is a diminution of the reflective quality of the mind, with resulting undue motor reaction. Under such circumstances the mental vigor can only be preserved by periods of absolute rest and change of occupation. It is especially in instances of morbid nervous mobility that the dietary plays an important part. Contrast the mild herbivora with the fierce carnivora, or further, consider the taming influence upon wild animals through withholding their meat. Where the nervous mobility is excessive, a non-nitrogenous diet will do much to restore normal mental action.

The value of habit in mental action cannot too strongly be insisted upon. Carpenter's dictum that "each part of the organism tends to form itself in accordance with the mode in which it is habitually exercised"[1] is especially true of the mind. The importance of this continuity of training is well shown in the easy eloquence of the orator, the fluency of the writer, the rapid disposal of vast commercial enterprises by the business man. Through habitual exercise of special faculties and their connecting tracts the action of the mind becomes more automatic and less dependent upon laborious attention.

The more completely the mind can exclude subjective impressions and become objective in its action, the more nearly can it attain to its fullest development. In the degenerative neuroses—hysteria, hypochondria and neuresthenia—we meet with subjective mental action in its most exaggerated form. The sufferer pays little attention to objective effort; his whole mental field is occupied with self-feeling, how much he suffers, what he dreads, often indeed, to the exclusion of the objective measures he must follow to successful recovery. He is ready to converse by the hour upon his fears, the exaggerated physical sensations, he imagines symptoms of incurable maladies and to turn his attention to objective effort is a herculean task. While these two antagonistic phases of mind are found alike in men and women, certainly subjective mental action is far more common among the latter. I venture to assert that this more than any other mental quality distinguishes the sexes. Whether it be due to education, environment or more sensitive nervous organisation, it certainly is true that as society is now constituted the feminine mind is more largely occupied with subjective impressions, feelings and ideas than man's. Her early training, home duties, society relations, all largely tend to heighten subjective attention. A man's whole training is in the direction of objective effort. His mental life deals with realities outside of self. Need we wonder, then, that a vast proportion of victims of these subjective neuroses are women whose mental horizon is so

1. Carpenter, Mental Physiology, p. 344.

largely subjective in character. What bearing this has upon the question of female suffrage and equal ability as well as liability with man, I will leave you to consider. Does it not emphasise, rather, the necessary duality of life, the complemental relations of the masculine and feminine mind?

The moral agencies which work together to fortify the mind are nowhere better expressed than in the beautiful epistle to the Corinthians in which St. Paul commends the Christian virtues "Faith, hope and love, and the greatest of these is love."

Belief, not doubt, leads up to success. Hope, not fear, kindles enthusiastic effort. Love, not hate, enriches and ennobles the human mind.

Suggestion and imitation also have much to do with the development of vicious mental action. Mental degeneration is a reversion to the infantile mind, the formative principle of which is imitation. The operation of these influences is met with among what may be termed "secondary degenerates," individuals whose mental action is largely imitative, not original—"following after strange gods." It is the curious contagion which dominates the mental action of mobs, of communities, of a populace.

Elam, in his interesting work, A Physician's Problem, wisely remarks: "There is a strong tendency for opinion, once promulgated, to run into an epidemic form. No opinion, no delusion is too absurd to assume the collective character."[1] The "imitative instinct," so-called by Hecker in his classical work, On the Epidemics of the Middle Ages, has at different periods of the world's history wrought fearful ravages in the mind of man. In religion, call to mind Peter the Hermit and the Crusaders; in finance, Law's bank and the Mississippi scheme in France; the South Sea bubble in England; Joseph Smith and Mormonism in America; and in our own day, the "silver craze."

This form of collective mentality is properly a matter for state medicine and deeply concerns the welfare of the public. In its prevention we must recognise the importance of legislative action quite as absolutely as we already accept rigidly restrictive laws for the prevention of diseases affecting the body. The influence of detailed publication in the daily prints of sensational cases of crime, of horrible accidents, of vice of every sort, has a deteriorating effect upon the public mind and should be checked by law, backed by public opinion. It is well known in medical jurispru-

1. Elam, page 190.

dence that sensational publicity of an unusual crime is at times followed by a series of crimes similar in character. More than one "Jack the Ripper" was concerned in the horrible mutilations so recently recorded. Within our own borders a late murder was ascribed to the suggestive influence of a newspaper clipping.

Our own state has done a great service to humanity in recognising this evil by prohibiting more than a brief statement of the facts of an electrocution.

It is not the publicity of facts we deplore : it is excitation of the baser emotions and passions of humanity by vivid and sensational distortion of fact we would see repressed. In other directions the state, by law, has checked, or in time will check, influences which weaken the mind and tend to its decay. Through factory and mining laws, through pure food laws, through the repression of intemperance, through proper penalties for crime due to dominating impulse, through the repression of vicious books, performances and brutalising shows, through education in its fullest sense much can be done and must be done for the protection of the defective mind.

Many a so-called crime is due to defective mental action, under the spur of suggestion. Many a criminal now serving out his sentence might justly accuse society of wronging him by ignoring the hygienic needs of the mind as it dare not those of the body. "Thou shalt not steal" has for its corollary, "lead us not into temptation." When we consider the limitations of the ignorant, the moral and mental defects of degeneracy and the scant provisions of law for the protection of the weaker mind, we may well join in the Arab's prayer, "Oh, God, be kind to the wicked : to the good thou hast already been sufficiently kind in making them good."

295 Alexander Street.

